# Complications of Hypertriglyceridemia in Pregnancy and Its Impact on Neonates: a Hospital-Based Study From Odisha

**DOI:** 10.7759/cureus.28399

**Published:** 2022-08-25

**Authors:** Sudarshan Dash, Malvika Tiwari, Putul Dash, Kaustav Kar, Nirmal K Mohakud

**Affiliations:** 1 Obstetrics and Gynecology, Kalinga Institute of Medical Sciences, Bhubaneswar, IND; 2 Obstetrics and Gynecology, Ramakrishna Mission Seva Pratishthan, Kolkata, IND; 3 Pediatric Medicine, Kalinga Institute of Medical Sciences, Bhubaneswar, IND

**Keywords:** pregnancy, neonates, preeclampsia, pancreatitis, hypertriglyceridemia

## Abstract

Objective

Hypertriglyceridemia (HTG) in pregnancy causes an increased risk for maternal and fetal complications. But, reports on the impact of HTG in pregnancy on maternal and fetal outcomes are scarce in developing countries. We aim to determine the maternal and neonatal complications of HTG in pregnancy.

Materials and methods

This prospective observational study was conducted on 150 pregnant women with HTG in the department of obstetrics and gynecology, KIMS, Bhubaneswar, from December 2019 to November 2020. Measurement of triglycerides during the first trimester, second trimester, and delivery was done. Maternal complications and neonatal outcomes in HTG mothers and mothers with normal triglyceride levels were compared.

Results

Out of 150 HTG cases, hypothyroidism, preeclampsia, acute pancreatitis, and sickle cell crisis occurred in 41 (27.3%), 22 (14.7%), six (4%), and three (2%) cases, respectively. The triglyceridemia (TG) levels raised from 133.7±48.2 mg/dl in the first trimester to 232.8±151.0 mg/dl in the third trimester. There is a significant increase in TG levels at the time of delivery compared to the first and second trimesters (p< .001). Out of 140 neonates, 30 (21.4%) were preterm, eight (5.7%) had intrauterine growth restriction (IUGR), and four (6.06%) were macrosomic. Intrauterine death, preterm, and macrosomia are significantly associated with maternal HTG compared to normal mothers (p < .032). All mortalities were due to acute pancreatitis (6; 4%) among mothers and four intrauterine fetal death.

Conclusion

There is a steady increase in TG levels in the successive trimesters of pregnancy. Gestational severe hypertriglyceridemia causes life-threatening complications. HTG-induced acute pancreatitis needs to be managed aggressively to prevent maternal death. Neonates of HTG mothers suffer from complications like prematurity, IUGR, and macrosomia.

## Introduction

Hypertriglyceridemia (HTG) has been associated with increased maternal and fetal adverse outcomes [1]. During pregnancy, maternal triglyceridemia (TG) increases linearly with gestational age [2,3]. Dietary indiscretion (increased ingestion of high glycemic food, processed food, additional calorie institution at random) is the usual clinical observation by us in Odisha. Further, a sedentary lifestyle leading to an increase in obesity in India and Odisha is well known [4].

A review of 59 articles on the impact of hypertriglyceridemia either before or during pregnancy, related to maternal complications (preeclampsia or gestational diabetes mellitus) and neonatal complications (preterm delivery, macrosomia, hypoglycemia, or intrauterine growth restriction) was analyzed. It showed the limited amount and great variability of the data to establish normal triglyceride ranges during the three trimesters of pregnancy. The authors indicate the need to carry out further research to determine risks and effective interventions before pregnancy in order to reduce maternal and neonatal morbidity and mortality [5]. Data were derived from the Amsterdam Born Children and Their Development (ABCD) cohort study on elevated lipid levels during late pregnancy-associated complications revealed pregnancy-induced hypertension (4.9%); preeclampsia (3.7%); preterm birth (5.3%); small for gestational age (9.3%); large for gestational age (9.3%); and child loss (1.4%) [6]. However, such data are lacking from Odisha state and the eastern part of India.

Therefore, the present prospective study was designed to document (i) TG levels in all trimesters of pregnancy, ii) HTG kinetics during the study, and iii) the effect of HTG on the outcome (pregnancy-related complications in mother and fetal outcome). This will help plan effective measures required preconceptionally or in early pregnancy for the reduction of maternal and neonatal morbidity and mortality among hypertriglyceridemic mothers.

## Materials and methods

This was a prospective observational study conducted at the Department of Obstetrics and Gynecology, Kalinga Institute of Medical Sciences (KIMS), Bhubaneswar, from December 2019 to November 2020. KIMS is a 2000-bedded not-for-profit teaching hospital that caters to cases from all the strata of the socio-economic groups and both urban and rural pregnant women in Odisha. All consecutive pregnant women for an antenatal checkup (ANC) at the outpatient department were included. A total of 150 pregnant women with HTG (defined as plasma TG levels above the 95th percentile for age) with written consent for the study were enrolled. Normal blood levels of TGs during pregnancy are 150 mg/dl or less. Pregnant women found to have TG levels > 150 mg/dl during any of the trimesters were included. TG during pregnancy of < 200 mg/dl is considered a mild rise and a level of > 200 mg/dl is considered a high rise in HTG. For the illiterate women, the study was explained to them in the local Odia language, and a sample was collected after taking their thumb impression on the consent form. We have taken consecutive sampling during the study period. We have not taken a comparison group in this study as pregnant women coming for normal check-ups were reluctant to give blood samples in each trimester. However, the same group of 150 pregnant women was evaluated and followed up for two weeks after the post-partum period. Patients with cholestasis, cholelithiasis, and drug-induced pancreatitis were excluded from the study. Measurement of lipid profile (cholesterol and triglycerides) in all trimesters of pregnancy was done. However, we analyzed the impact of TG levels on maternal and neonatal outcomes. Detailed clinical profiles of the mothers and the babies were collected. The complete blood count (CBC) test, liver function test (LFT), thyroid function test, and renal function test were done. These tests were done to know the hematological and biochemical changes occurring during the course of pregnancy. The study was conducted according to the principles of the Helsinki Declaration upon approval by the research ethics committee of KIMS (KIIT/KIMS/IEC/232/2020).

Definitions

Rise of blood pressure > 140/90 mmHg was noted two times in a 6-hour difference and proteinuria after 20 weeks of pregnancy but resolved up to 12 weeks postpartum in a known normotensive woman, which is called preeclampsia [7]. Pathological increase of amniotic fluid volume in pregnancy is called polyhydramnios [8]. The World Health Organization (WHO) recommends a risk-based screening with a 75 gm 2-hr oral glucose tolerance test with fasting blood glucose between 92 and 125 mg/dL and 2-hr blood glucose 153-199 mg/dL to be taken as diagnostic for gestational diabetes mellitus (GDM) [9]. Placental abruption is the early separation of the placenta from the lining of the uterus before the completion of the second stage of labor [10]. Prelabor rupture of membranes (PROM) is defined as rupture of membranes before the onset of labor. When the membrane ruptures before 37 completed weeks of pregnancy, it is called preterm PROM (PPROM) [10]. Obstetric cholestasis is characterized by pruritus, raised serum aminotransferases (more than twofold), bile acid levels in the second or third trimester, and resolution of signs and symptoms post 2-3 weeks of delivery [11]. Live babies born after 28 weeks of gestation but, prior to 37 completed weeks of pregnancy are called preterm babies [12]. A baby who dies after 28 weeks of pregnancy, but before or during birth, is classified as a stillbirth [12]. Neonatal sepsis refers to blood culture-positive infection in neonates less than 28 days of life [12].

Statistical analysis

The data collected were entered into a Microsoft Excel form (Microsoft Corporation, Redmond, WA) for analysis, and descriptive statistics were calculated. Maternal and neonatal complications in gestational HTG were compiled. Besides, complications in mild and high maternal triglyceride levels of gestational HTG were compared with an unpaired t-test. SPSS Version 20 (IBM Corp., Armonk, NY) was used to do the statistical analysis of the data obtained in the study. p-value <0.05 was considered statistically significant.

## Results

Out of 150 pregnant women enrolled in the study, 140 underwent delivery at KIMS. The mean age of the women was 29.8 ± 2.8 years. The majority of them are from an urban background (n=81, 54%). The clinical profile and complications of pregnant women are described in Table 1.

**Table 1:** Maternal complications with HTG during pregnancy and delivery, n=150 HTG, Hypertriglyceridemia; * TSH value more than 2.5 mIU/liter in the first trimester, greater than 3 mIU/L in the second and third trimesters; #, diagnosed by ultrasonography and clinical picture; ¶, diagnosed clinically and serum bilirubin > 2 mg/dl or raised liver enzymes by twofold during pregnancy or the 2-week post-partum period

Parameters	Frequency (n=150)	Percentage (%)
Hypothyroidism*	41	27.3
Preeclampsia	22	14.7
Acute pancreatitis^#^	06	4.0
Jaundice^¶^	07	4.7
Polyhydramnios	03	2.0
Gestational diabetes mellitus	04	2.7
Acute fatty liver disease	03	2.0
Systemic inflammatory response syndrome	05	3.3
Abruptio placentae	03	2.0
Ascites	05	3.3
Sickle cell crisis	03	2.0
Preterm premature rupture of the membranes	03	2.0
Obstetric cholestasis	05	3.3
Death	6	4.0

A large number of cases (41, 27.3%) were detected to have hypothyroidism. Preeclampsia was detected in 22 (14.7%) cases. HTG-induced acute pancreatitis was found in six (4%) cases.

**Figure 1:**
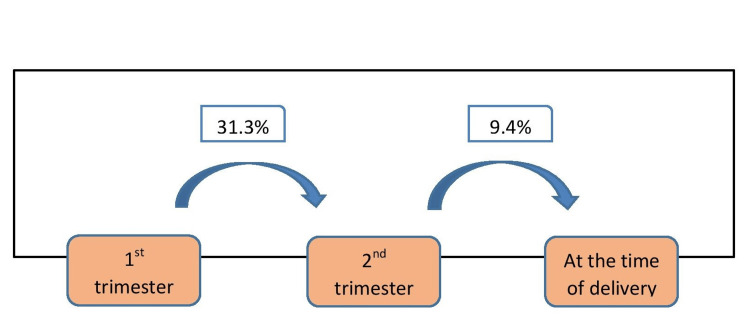
Increase in triglyceride (TG) levels in successive trimesters of pregnancy and during delivery

It was found that TG levels increase significantly from the first trimester to the second trimester by 31.3% (Figure 1).

**Figure 2:**
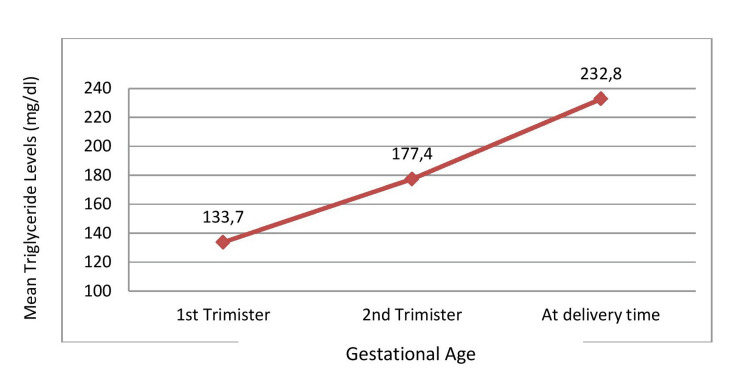
Increase in mean triglyceride levels (mg/dl) from the first trimester of pregnancy till delivery

Also, there was a steady increase in TG levels in successive trimesters of pregnancy (Figure 2).

Further, it was found that the severe grade of HTG in the first trimester was 0 and increased to nine (6.0%) by the time of delivery.

Comparison between the TG levels in different trimesters of pregnancy showed there was a rise in TG levels at the time of delivery compared to the first and second trimesters (p< .001) (Table 2).

**Table 2:** Comparison between the TG Level in different trimesters of pregnancy, n=140 TG, Triglycerides

	TG Level (mg/dl)Mean± S.D.	p-value
1st trimester(8-12 weeks) (a)	133.7±48.2	<0.001
2nd trimester(28-32weeks) (b)	177.4±86.2	
At the time of delivery (c)	232.8±151.0	
	Mean Difference	p-value
2^nd ^- 1^st^ trimester (b-a)	43.6	<0.001
At the time of delivery - 2^nd^ trimester (c-b)	55.5	<0.001
At the time of delivery - 1^st^ trimester (c-a)	99.1	<0.001

**Figure 3:**
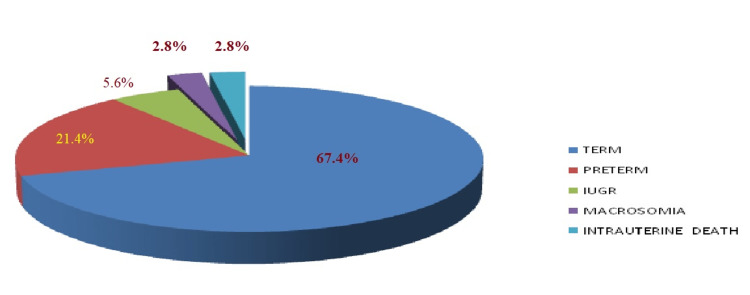
Characteristics and outcomes of neonates born to mothers in the study group, n=140 IUGR, Intrauterine growth restriction

Out of 140 neonates, 30 (21.4%) cases were preterm and eight (5.6%) were IUGR. Intrauterine death in four (2.8%) babies was associated with severe HTG (Figure 3).

Intrauterine death, preterm, and macrosomia are significantly associated with the high rise in maternal HTG compared to the mild rise in HTG mothers (p < .032) (Table 3).

**Table 3:** Neonatal outcomes in mild and high maternal triglyceride levels during delivery, n=140 *, Percentages

Neonatal outcomes	Triglyceride levels		p-value
	Mild rise (n=74)^*^	High rise (n=66)*	
Intrauterine death	0	4 (6.1%)	0.032
Macrosomia	0	4 (6.1%)	0.032
Intrauterine growth restriction	3 (4.1%)	5 (7.5%)	0.375
Preterm	4 (5.4%)	13 (19.7%)	0.010

A comparison of complications in gestational high-rise TG with the mild-rise TG level showed that acute pancreatitis, GDM, and sickle cell crisis were strongly associated with severe hypertriglyceridemia (p < .05) (Table 4). Though hypothyroidism, preeclampsia, and pregnancy-induced altered liver function (jaundice, acute fatty liver, and obstetric cholestasis) were frequently associated with HTG in pregnancy, they were statistically not significant in comparison with the mild-rise TG level in pregnant women.

**Table 4:** Comparisons of maternal complications in mild hypertriglyceridemia Vs. high triglyceride levels, n=150

Complications in pregnancy	Triglyceride levels		p-value
	Mild rise (n=80)	High rise (n=70)	
Preeclampsia	12 (15.1%)	10 (14%)	0.848
Hypothyroidism	21 (26.5%)	20 (28.1%)	0.828
Jaundice	3 (3.7%)	4 (5.6%)	0.594
Polyhydramnios	2 (2.4 %)	2 (2.8%)	0.898
Gestational diabetes mellitus	0	4 (5.6%)	0.032
Premature rupture of membranes	1 (1.2%)	2 (2.8%)	0.498
Acute fatty liver of pregnancy	1 (1.2%)	2 (2.8%)	0.498
Systemic inflammatory response syndrome	1 (1.2%)	4 (5.6%)	0.121
Abruptio placentae	2 (2.5%)	1 (1.4%)	0.624
Ascites	1 (1.2%)	4 (5.6%)	0.137
Sickle cell crisis	0	3 (4.2%)	0.049
Pancreatitis	0	6 (8.4%)	0.009
Obstetric cholestasis	1 (1.2%)	4 (5.6%)	0.137

## Discussion

In this study, we highlighted the association of gestational hypertriglyceridemia with maternal and fetal complications. The study found maternal complications like pancreatitis with severe hypertriglyceridemia (p < .009). A study on 116,550 Danish subjects revealed that events of pancreatitis are 5.5 per 10,000 person-years, which is quite low compared to our cases [13]. This might be due to the bias in selecting HTG cases only in our study. Besides, sickle cell crisis and GDM are strongly associated with hypertriglyceridemia (p< 0.05). Also intrauterine death, preterm, and macrosomia are significantly associated with maternal HTG compared to normal mothers (p < .032).

In pregnancy, there is an estrogen-induced altered lipid metabolism, so that the fetus can get adequate transfer of nutrients through the placenta. A few women having a genetic association or a secondary precipitating factor may have severe gestational HTG. It can cause many life-threatening complications like acute pancreatitis [13,14]. It is suggested that triglyceride-rich lipoproteins promote lipolysis resulting in an increased level of free fatty acids (FFAs), which causes injury to acinar cells and the vascular endothelium of the pancreas. Further, in-vitro studies found fatty acid-induced mitochondrial injury due to hypertriglyceridemia. The acidic environment by ischemia increases the toxicity of the FFAs and causes more pancreatic injury [15]. A diagnosis of acute pancreatitis can be made by the presence of two of the three criteria, i.e., severe acute epigastric pain; > 3 times rise of serum amylase or lipase; pancreatitis in imaging examinations [16]. Acute pancreatitis in pregnancy can occur during any trimester, but >50 % of cases are found in the third trimester and the post-partum period. The presence of gallstones is commonly associated with acute pancreatitis [8]. We have found six (4%) cases of acute pancreatitis, all of which were died. This may be due to late presentation to the hospital and inappropriate management strategies as there is a lack of awareness/suspicion among treating obstetricians. Severe gestational HTG should ideally be managed with a low-fat diet (fat should not be more than 20% of total calories), medium chain TG oil, fibrates, statins, and fish oil. If there is no improvement with the above measures like total parenteral nutrition with intravenous omega-3 supplementation, weekly plasmapheresis is the treatment of choice [13].

We found severe HTG in six cases out of 150 cases. All these cases have secondary causes like obesity, hypothyroidism, and multipara. Preeclampsia is found in 10 (14%) cases among gestational HTG, which is more compared to 1-4% in general pregnancy cases in India [17]. However, a study in Cape Coast Metropolis found preeclampsia in 8% of HTG cases [18]. Preeclampsia in HTG could be explained by various mechanisms like HGT-induced endothelial injury by oxidative stress and decreasing prostacyclin in endothelial cells or insulin resistance due to gestational HTG in pregnancy. The FFA/albumin ratio is increased in women with preeclampsia leading to further lipolytic activity, thereby more endothelial FFA uptake and esterification to TG [2]. The previous study shows an increase in proatherogenic lipids like TG, small dense low-density lipoprotein (LDL), and decreased high-density lipoprotein (HDL); with progression, pregnancy may lead to preeclampsia [19,20].

GDM is significantly associated with cases of gestational HTG in this study group. A previous study found the association between maternal abdominal obesity and HTG in the first trimester has a greater propensity for glucose intolerance as pregnancy proceeds [21]. A study by Wang et al. found that resistance to insulin, decreased secretion of insulin, and oxidative stress in pregnancy contributed mostly to maternal HTG and GDM in pregnancies [22].

A large number of cases (n=15, 10%) have altered liver function and clinically manifested as obstetric cholestasis, jaundice, and acute fatty liver disease. Intrahepatic cholestasis during pregnancy may cause an increased risk of preterm delivery and fetal morbidities [23]. So, in this type of situation, induction of delivery is to be done early to prevent fetal complications [23].

We found that a high rise in TG in pregnancy leads to a sickle cell crisis compared to low-rise TG mothers (p< 0.05). A study (Suzana Z et al. 2011) had shown that elevated TG correlates significantly with markers of hemolysis, endothelial activation, and inflammation [24]. This might be the reason for the sickle cell crisis in HTG mothers. Sickle cell disease (SCD) in pregnancy may cause pre-eclampsia, abortions, premature delivery, and IUGR; appropriate measures during pregnancy lead to better outcomes. This may be a confounding factor in our study, however, the SCD population in this group accounts for 2% of cases only. The recent development in preimplantation genetic assessment and prenatal diagnosis are used to counsel couples with SCD for a normal baby [25].

Preterm and IUGR babies accounted for 27.24% of delivered neonates from HTG mothers. This may be due to HTG-induced preeclampsia contributing to the increased number of spontaneous delivery of preterm births. Also increased TG level leads to a greater risk for large-for-gestational-age babies [19]. In this group, we found that 5.6% of neonates have macrosomia, which is significantly higher compared to high TG level mothers to lower TG level mothers (p < 0.032).

We have reported four neonatal deaths and all are out of mothers with acute pancreatitis. All babies died due to prematurity and neonatal sepsis. All the mothers with acute pancreatitis presented late in our obstetric unit. So, early detection and aggressive management of severe HTG in acute pancreatitis are needed. For that, awareness among obstetricians of clinical suspicion of acute pancreatitis is required. Besides, a team of neonatologists is required to take care of these premature babies in a separate isolation level-3 NICU, as most of these neonates are referred cases from peripheral hospitals and are suspected to have sepsis.

This study revealed a progressive rise in plasma triglyceride levels from the first trimester (133.7±48.2 mg/dl) to the third trimester (232.8±151). It was found that plasma lipids increase after the first trimester of pregnancy, but significant increases in TG levels (2-fold) occur by the third trimester (mean TG levels 0.89 mmol/L, 1.71 mmol/L, and 2.77 mmol/L in the first, second, and third trimesters, respectively) [2,26]. In a study by Pusukuru et al., 2009, the mean TG levels found in the second and third trimester of pregnancy were 188.68±20.88 mg/dl and 216.78±20.09 mg/dl, respectively, which is corroborative of our findings [27].

To prevent fetal and maternal complications, treatment for gestational HTG should be initiated early and aggressively [6]. For that, a multidisciplinary team including an obstetrician, endocrinologist, neonatologist, gastroenterologist, and dietician is needed. Moreover, motivated counseling at the first detection of HTG in the early stage of pregnancy in the outpatient department for an appropriate fat-restricted diet should be done [4]. Furthermore, early induction of delivery in near-term pregnancy with the skilled and equipped NICU team for the management of preterm babies is desired for the best possible outcome for mothers and babies.

Limitations

This is a single-centered study with small sample size. Pre-conceptional measurement of lipid levels could have given better insight for early detection, but we have taken serial estimation of TG in all trimesters to know the progression of TG levels and associated complications. We have lost a few patients till delivery, as they had delivered in other hospitals. We have not taken any pre-pregnancy lipid levels of the cases to exclude hyperlipidemia. Also, we have not taken a comparator group of normal pregnant cases in this study. Other parameters of the lipid profile like serum cholesterol are not analyzed, as we have focused on the association of TG levels with maternal and fetal complications in this study.

## Conclusions

There is a progressive rise in TG levels as pregnancy advances and it is almost twofold by the time of the third trimester. Gestational severe hypertriglyceridemia is associated with life-threatening complications like preeclampsia, pancreatitis, sickle cell crisis, preterm delivery, IUGR, and fetal death. HTG-induced acute pancreatitis may cause maternal and fetal death if not detected early and managed aggressively. Neonates of HTG mothers may have more complications like prematurity and IUGR, needing a trained NICU team ready at the time of delivery.
